# Editorial: Cutaneous immunology

**DOI:** 10.3389/fmed.2023.1275332

**Published:** 2023-08-24

**Authors:** Gianluca Avallone, Carlo Alberto Maronese, Angelo Valerio Marzano

**Affiliations:** ^1^Dermatology Clinic, Department of Medical Sciences, University of Turin, Turin, Italy; ^2^Dermatology Unit, Fondazione IRCCS Ca' Granda Ospedale Maggiore Policlinico, Milan, Italy; ^3^Department of Pathophysiology and Transplantation, University of Milan, Milan, Italy

**Keywords:** cutaneous immunology, dupilumab, bullous pemphigoid, prurigo nodularis, immunotherapy, alopecia areata, inflammation

The skin represents the outermost organ of the body and is continuously exposed to external pathogens. Alongside its recognized protective function, the skin harbors a complex and dynamic immune system. It encompasses an intricate immune network comprising distinct resident immune cells, such as Langerhans cells, keratinocytes, B and T lymphocytes, and macrophages (1, 2).

Advancements in cutaneous immunology research have led to a transformation in the landscape of dermatological treatments. In recent years, a deeper understanding of the underlying immune pathways has further paved the way for the development of biologics and small-molecule inhibitors that precisely target key molecules involved in skin inflammation.

A paradigmatic example of the critical role of cutaneous immunology is evident in prurigo nodularis. Since marked and lasting responses may not always be achievable from available treatments, this condition can represent a complex clinical *scenario*. Significant progress has been made in understanding the pathophysiology of itch, with increasing details emerging regarding the underlying mechanisms (3). An upregulation of IL-4 and signal transducer and activator of transcription 6 expression has been found in skin lesions of patients with prurigo nodularis (4), leading to the consideration of dupilumab as a potential therapeutic option in this condition (5). Cao et al. reported intriguing findings that highlight the effectiveness of dupilumab in prurigo nodularis, even in patients who had previously undergone multiple therapies. These real-life data further support the outcomes observed in recent phase III trials, providing evidence that targeting the IL-4 and IL-13 axis with dupilumab is an effective and safe approach for managing prurigo nodularis (6).

Recent research has provided insights into the distinct pathways through IL-4 and IL-13 exert their effects (7). According to Napolitano et al. IL-13 not only recruits inflammatory cells but also modulates the cutaneous microbiome and influences the skin barrier by affecting the expression of barrier proteins. The enhanced understanding of the diverse functions of this interleukin has driven the development of targeted therapies, such as tralokinumab and lebrikizumab, which have shown significant efficacy in reducing atopic dermatitis severity (8, 9).

Advancements in cutaneous immunology also encompass additional molecular pathways, involving several other pathologies. As extensively detailed in the review article by Xu et al. promising therapeutic targets are emerging for alopecia areata. The article provides a comprehensive overview of the evolving insights into the roles of specific cell populations in the pathogenesis of this condition. Tramontana et al. thoroughly review the pathophysiological mechanisms underlying contact dermatitis, along with the identification of newly recognized allergens. This newly acquired understanding opens up opportunities for developing emerging therapeutic approaches based on this knowledge.

Activation of the cutaneous immune system can occur not only during inflammatory pathologies but also through immunotherapy, leading to diverse implications (10). Immunotherapy can offer significant advantages for patients, as extensively reported in the management of melanoma (11). In recent years, this has led to a debate aimed at understanding which patient group benefits more from immunotherapy (12). Jaeger et al. attempted to address this question through a meta-analysis, revealing that NRAS-mutant cutaneous melanoma demonstrated an increased likelihood of a partial or complete tumor response compared to NRAS-wildtype cutaneous melanoma. Therefore, genomic screening for NRAS mutations in patients with metastatic melanoma could enhance predictive ability when initiating immunotherapy.

In patients receiving immune checkpoint blockade inhibitors can occurr an excessive and non-specific immune system activation with the potential to affect various organs (13). The skin is commonly involved, with around 30% of patients treated with anti-PD-L1 drugs experiencing skin manifestations (14). This encompasses a wide spectrum of possible cutaneous presentations, including immunotherapy-associated autoimmune bullous dermatoses (15). The study conducted by Merli et al. provides insights into the Italian experience with these entities and reviews the current literature on the topic. Interestingly, they observed that immunotherapy-induced pemphigoid seems to have a male predominance, an earlier onset age compared to the classic variant, and a potentially longer prodromal phase.

In order to achieve an accurate diagnosis, it is crucial be aware that certain immune-mediated disorders can show atypical clinical manifestations, which may result in the misdiagnosis of underlying condition. This aspect was well demonstrated in a case-series conducted by Le et al. where four patients with pemphigus vulgaris presented with a rare manifestation defined as “mounded and refractory keratoses”. This particular presentation is characterized by scaling plaques on the scalp and trunk, often observed in individuals with skin of color and exhibiting distinctive features different from pemphigus vegetans. Awareness and recognition of this variant may assist healthcare providers in the diagnosis of pemphigus vulgaris and may lead to timely initiation of appropriate therapy.

In conclusion, cutaneous immunology has made remarkable progress in its comprehension. As our Research Topic has explored, this not only holds theoretical significance but also bears various practical implications, as it is enabling the development of novel drugs and a better elucidation of both common and rare clinical entities. In the future, a more comprehensive understanding is warranted, and we hope that this Research Topic will stimulate further basic, translational, and clinical research in the field.

## Author contributions

GA: Conceptualization, Writing–original draft, Writing–review and editing. AM: Writing–review and editing. CM: Conceptualization, Writing–review and editing.
